# An On-Chip Microscale Vacuum Chamber with High Sealing Performance Using Graphene as Lateral Feedthrough

**DOI:** 10.3390/mi14010084

**Published:** 2022-12-29

**Authors:** Panpan Yu, Fangyuan Zhan, Weidong Rao, Yanqing Zhao, Zheng Fang, Zidong Tu, Zhiwei Li, Dengzhu Guo, Xianlong Wei

**Affiliations:** 1 Hunan Institute of Advanced Sensing and Information Technology, Xiangtan University, Xiangtan 411105, China; 2 School of Electronics, Peking University, Beijing 100871, China

**Keywords:** on-chip, microscale vacuum chamber, graphene, lateral feedthrough, Paschen’s law

## Abstract

On-chip microscale vacuum chambers with high sealing performance and electrical feedthroughs are highly desired for microscale vacuum electronic devices and other MEMS devices. In this paper, we report an on-chip microscale vacuum chamber which achieves a high sealing performance by using monolayer graphene as lateral electrical feedthrough. A vacuum chamber with the dimensions of π × 2 mm × 2 mm × 0.5 mm is fabricated by anodically bonding a glass chip with a through-hole between two Si chips in a vacuum, after monolayer graphene electrodes have been transferred to the surface of one of the Si chips. Benefiting from the atomic thickness of monolayer graphene, the leak rate of Si–glass bonding interface with a monolayer graphene feedthrough is measured at less than 2 × 10^−11^ Pa·m^3^/s. The monolayer graphene feedthrough exhibits a minor resistance increase from 22.5 Ω to 31 Ω after anodic bonding, showing good electrical conductance. The pressure of the vacuum chamber is estimated to be 185 Pa by measuring the breakdown voltage. Such a vacuum is found to maintain for more than 50 days without obvious degradation, implying a high sealing performance with a leak rate of less than 1.02 × 10^−16^ Pa·m^3^/s.

## 1. Introduction

Micro-Electro-Mechanical System (MEMS) devices have attracted much attention due to their compact size and low cost. Many MEMS devices, such as gyroscopes [1], RF devices [2], and vacuum devices [3], require isolation of their key components against dense gas molecules or contamination from the environment. Therefore, a microscale vacuum chamber needs to be constructed with some bonding technologies, where a fine electrical feedthrough connecting the electrical component in a MEMS vacuum chamber with the circuits outside is of great importance.

Electrical feedthroughs in MEMS devices can be summarized as being in two classes: vertical feedthrough and lateral feedthrough. Vertical feedthrough usually achieves a conducting channel by drilling a via-hole through a glass [4] or silicon [5] substrate and filling it with metal materials. Torunbalci, MM [6] used two separate SOI wafers to fabricate highly doped TSV and suspended MEMS structures, achieving a chamber with a vacuum of around 1 Torr. However, vertical feedthroughs generally require complex and high-cost micro-nano processes, which hinder their widespread application. As for lateral feedthrough, where a metallic electrode is evaporated on the substrate by micro-processing technology, they face the problem of step-like gas leakage channels caused by the thickness of the electrode. When the metal electrode is 75 nm, the air leak rate at the interface was 8 × 10^−8^ Pa·m^3^/s [7]. To ensure a high airtightness, the thickness of the lateral metal electrode is strictly limited, which limits the conductance of the feedthrough as well. An alternative lateral feedthrough is the embedded structure [8], which requires a channel etched for filling with metallic electrode. However, embedded structure also requires complex and high-cost micro-nano processes, and its sealing performance is far from satisfactory [9]. An easy way to realize a microscale vacuum chamber with a fine electrical feedthrough and a high sealing performance is still highly desired.

Graphene, a one-atom-thick nanomaterial, has been of wide interest in recent years [10]. Its monolayer structure [11] and remarkable electrical conductivity make it an ideal electrode material [12] for lateral feedthrough in MEMS devices. In this paper, we report an on-chip microscale vacuum chamber with monolayer graphene as lateral feedthrough. The on-chip microscale vacuum chamber is found to exhibit lateral feedthrough with good electrical conductance and an excellent sealing performance. This provides a convenient way to realize a high-performance vacuum chamber for MEMS devices.

## 2. Device Structure and Processing

**Figure 1:**
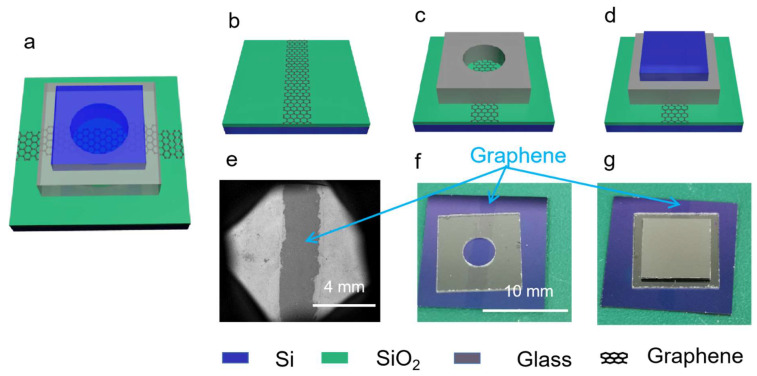
Device structure and fabrication processes. (**a**) Schematic structure of the device; (**b**) Transferring and patterning graphene; (**c**) Two-layer device after first anodic bonding; (**d**) Three-layer device after second anodic bonding; (**e**) An SEM image of the structure in (**b**); (**f**) A photograph of the structure in (**c**); (**g**) A photograph of the structure in (**d**).

A sandwich-like on-chip microscale vacuum chamber was constructed by anodic bonding. The schematic structure of the device and its fabrication processes are shown in Figure 1.

A 15 mm × 15 mm Si/SiO_2_ (500 µm/300 nm) chip was first treated by reactive ion etching (RIE) with O_2_ plasma for hydrophilicity [13]. Then, a 3 mm × 15 mm sheet of monolayer graphene, purchased from the Beijing Graphene Research Institute, was transferred to the Si/SiO_2_ chip, as shown in Figure 1b. It can be clearly seen in a scanning electron microscope (SEM) that a graphene electrode with dark contrast lies in the middle of the Si/SiO_2_ chip (Figure 1e). In the process of transferring the graphene electrode, we innovatively introduced a blue PE protective film as the mask for dry etching, without using photolithography technology, which simplified the fabrication process and reduced the cost.

To construct a microscale vacuum chamber, two subsequent anodic bonding processes were conducted. The first anodic bonding was performed between the Si/SiO_2_ chip with a graphene electrode and a 10 mm × 10 mm × 0.5 mm BF33 glass spacer with a through-hole of diameter 4 mm in the middle. The anodic bonding took place at 430 °C at a vacuum level of about 7 × 10^−5^ Pa, where a voltage of about 1400 V was applied on the Si/SiO_2_ chip for about 135 min. The electrical current during bonding rapidly rose to the highest point 0.023 mA, and then gradually decreased to 0 mA. The schematic structure and a picture of the device after first anodic bonding are shown in Figure 1c,f, where it can be clearly seen that a graphene electrode runs through the glass spacer at the Si/SiO_2_–glass interface.

The second anodic bonding was performed between the two-layer device after the first anodic bonding and an 8 mm × 8 mm × 500 µm Si chip, so that the opening was closed. The second bonding was performed using the same procedure as the first, yet the bonding time was only about 45 min. An on-chip microscale vacuum chamber was thus achieved, as shown in Figure 1d,g.

## 3. Results and Discussion

### 3.1. Characterization of Graphene Electrode

In this work, monolayer graphene was used as lateral feedthrough passing through the Si/SiO_2_–glass bonding interface.

**Figure 2:**
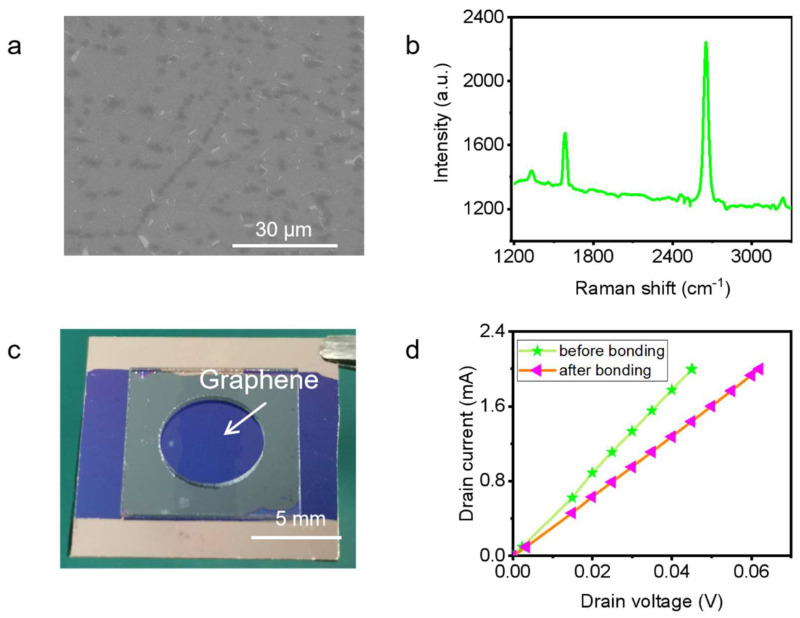
Characterization of graphene electrode. (**a**) An SEM image of the graphene after transferring; (**b**) Raman spectrum of monolayer graphene; (**c**) A photograph of measuring the conductance of graphene electrode; (**d**) *I*−*V* curves of graphene electrode before and after bonding.

The morphology of the graphene electrode after transferring was determined by SEM, as shown in Figure 2a, showing that the graphene has been successfully transferred to the Si/SiO_2_ chip without obvious damage. The structure of the graphene used here was determined by Raman spectroscopy [14,15,16], as shown in Figure 2b. The measurement was performed at room temperature at 633 nm wavelength. It can be obviously observed that the G peak is at about 1580 cm^−1^ and the 2D peak is at about 2700 cm^−1^, and the intensity of the 2D peak is significantly higher than that of the G peak. In addition, the 2D peak is symmetrical and sharp. These confirm that the graphene used in this paper is monolayer.

The conductance of the graphene electrode should be characterized since the anodic bonding was performed under the conditions of high temperature and high voltage. To obtain a precise conductance of the graphene electrode, Ti/Au electrodes with the thickness of 5 nm/40 nm were deposited on both ends of the graphene electrode before anodic bonding to achieve a good electrical contact (Figure 2c).

As shown in Figure 2d, the *I*–*V* curves of the graphene electrode before and after bonding were measured to study the effect of anodic bonding on the conductance of the graphene electrode. The measurements were performed in a probe station (Janis ST-500-1-UHT-(4TX)) with a semiconductor characterization system (Keithley 4200). It can be seen from the image that both of the *I*–*V* curves have a good linear relationship. The resistance of the graphene electrode is 22.5 Ω before bonding and 31 Ω after. Such a resistance is better than many other devices using lateral [17] or vertical feedthroughs [18,19,20], which means it is good enough for a variety of applications like sensors. In conclusion, the conductance of the graphene electrode did not experience much degradation after anodic bonding. Therefore, it is feasible for an on-chip microscale vacuum chamber sealed by anodic bonding to use monolayer graphene as a lateral feedthrough.

### 3.2. Airtightness of Si–Glass Bonding Interface

On-chip microscale vacuum chambers often require high sealing performance, so airtightness of bonding interface is a very important issue. The bonding interface’s airtightness with respect to the two-layer device shown in Figure 1f is first characterized.

**Figure 3:**
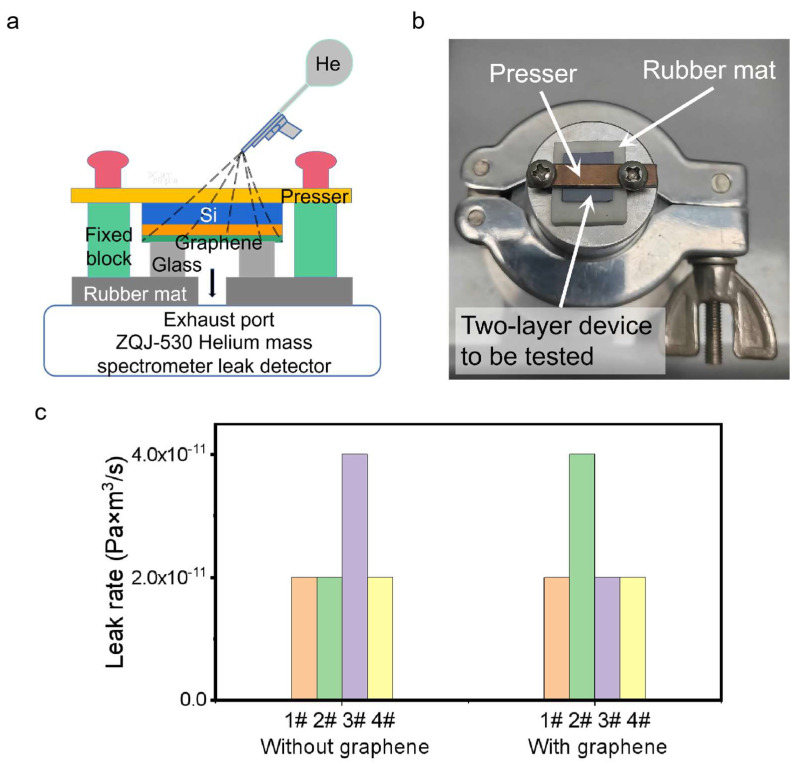
Sealing performance of two-layer device. (**a**) Schematic of a helium mass spectrometer leak detector; (**b**) A photograph of the helium mass spectrometer leak detector; (**c**) Comparison of leak rates between devices without and with graphene (each colored bar represents one device).

A ZQJ-530 Helium Mass Spectrometer Leak Detector was used to test the airtightness of the bonding interface. As shown in Figure 3a,b, a two-layer device with a graphene electrode through the Si/SiO_2_–glass bonding interface (Figure 1f) caps the exhaust port of the leak detector. A rubber mat with a through-hole in the middle is sandwiched between the device and the exhaust port to keep their interface tight. After the leak detector starts to work, the chamber of our apparatus is pumped to vacuum. When a small amount of helium gas is sprayed near the bonding interface outside the chamber, we can read the leak rate of the bonding interface. The detection limit of the leak detector is 2 × 10^−11^ Pa·m^3^/s.

As shown in Figure 3c, the leak rates of eight two-layer devices without/with graphene electrode at the bonding interface are determined. Three devices without graphene electrode have leak rates of 2 × 10^−11^ Pa·m^3^/s and the leak rate of the other one is 4 × 10^−11^ Pa·m^3^/s. In comparison, four devices with graphene electrodes as lateral feedthroughs have similar leak rates, with three at 2 × 10^−11^ Pa·m^3^/s and the other at 4 × 10^−11^ Pa·m^3^/s. The detection limit of the leak detector has been reached within the allowable range of the error of the instrument itself, indicating that leak rate of devices using monolayer graphene electrode as lateral feedthrough is generally better than 2 × 10^−11^ Pa·m^3^/s, which is much more satisfactory than when using metal electrode [7].

### 3.3. Sealing Performance of Microscale Vacuum Chamber

As the pressure in a microscale vacuum chamber as small as several cubic millimeters is difficult to measure directly, we developed a method using Paschen’s law to approximately evaluate the vacuum level and the leak rate of a three-layer device shown in Figure 1g.

Paschen’s law [21] describes the electric discharge voltage between two conductive materials as a function of the gap distance *d* (m) and the pressure *P* (Pa) of the intervening gas. For large gaps (*d* > 100 μm [21]), the “Paschen curve” describes the breakdown voltage *V_B_*(V) as

(1) $$V_{B}=\frac{BPd}{ln\left(APd\right)-ln\left[ln\left(1+\frac{1}{\gamma }\right)\right]},$$

where *A* and *B* are two fitting parameters, *γ* is the second Townsend coefficient which describes the mean number of generated secondary electrons per ion. Parameters *A* = 10.95 Pa^−1^m^−1^, *B* = 273.78 VPa^−1^m^−1^ in air are well determined by ref [22]. Equation (1) predicts very high breakdown voltages for very small products of *Pd*. As *Pd* increases, *V_B_* first decreases to a minimum at several hundred Volts and then increases again. At low pressures, few collisions require high probability of ionization, so a higher electric field is needed. At high pressures, short mean free path makes it difficult for electrons to accumulate enough energy to reach ionization energy, which also requires a higher electric field, resulting in a minimum in Paschen’s law.

We first prepared a three-layer device by stacking Si–glass–Si chips in the air without bonding, with the sizes of the glass spacer and the Si chips the same as those described above. The gap between electrodes in our configuration is 0.5 mm, which is well above the microscale boundary where Paschen’s law may deviate due to field emission [23]. The measured breakdown voltages of the device were 2100 V, 2200 V, 2260 V, 2210 V, respectively, for four measurements, in which the average value of *V_B_* was 2192.5 V. Taking *P* = 1.01 × 10^5^ Pa, *d* = 0.5 mm, *A* = 10.95 Pa^−1^m^−1^, *B* = 273.78 VPa^−1^m^−1^, *V*_B_ = 2192.5 V, the second Townsend coefficient is calculated as 0.57 according to Equation (1). Therefore, the parameter values of Paschen’s law are *A* = 10.95 Pa^−1^m^−1^, *B* = 273.78 VPa^−1^m^−1^, *γ* = 0.57, which will next be used to estimate the vacuum level of a three-layer device with a microscale vacuum chamber.

**Figure 4:**
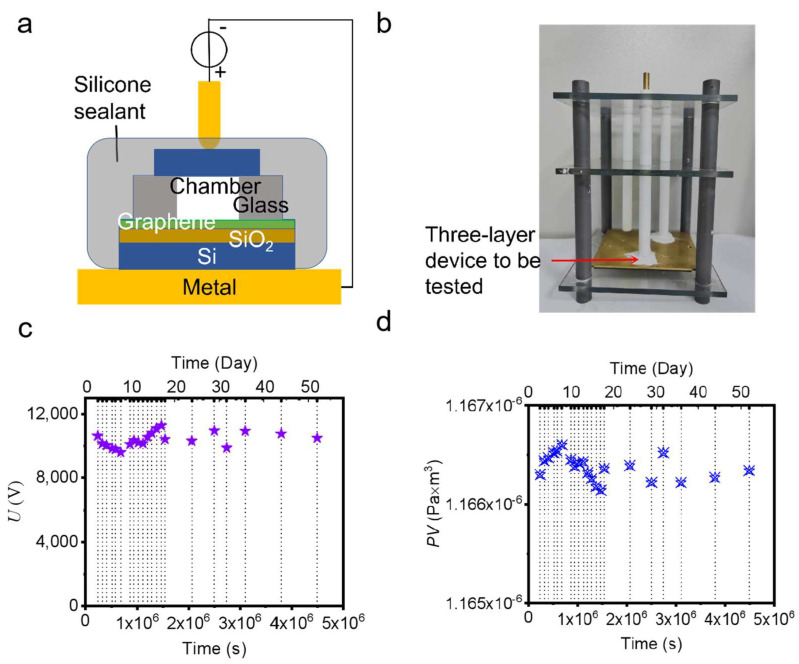
Sealing performance of three−layer device. (**a**) Schematic of a test apparatus; (**b**) A photograph of the apparatus; (**c**) Changes of the breakdown voltage of the three−layer device with time; (**d**) Changes of the *PV* of the three−layer device with time.

A simple apparatus was developed to test the breakdown voltage of our device, as shown in Figure 4a. During the breakdown test, a three-layer device to be tested (shown in Figure 1g) is fixed between a metal plate and a metal rod, and then a silicone sealant is coated around them and dried to isolate the tested device from the external environment to avoid unplanned discharge from outside. A high voltage is applied on the top rod and the plate at the bottom is connected to ground. Figure 4b is a photograph of such an apparatus.

Finally, a microscale vacuum chamber device using a graphene electrode as lateral feedthrough with a high sealing performance was fabricated (shown in Figure 1g). The breakdown voltage measured with time is shown in Figure 4c, fluctuating around 10,400 V for more than 4 × 10^6^ s (50 days) within the allowable range of errors. The breakdown voltage corresponds to a pressure of the chamber of 185 Pa according to Paschen’s law with the above determined parameters. Considering the flow resistance caused by our bonding apparatus and the residual gas generated in the bonding process and released from inner surfaces, such a vacuum level is reasonable [24] and can be improved by adding a movable component to the equipment or adding a getter [25] to the chamber, respectively. As the dimensions of the vacuum chamber are π × 2 mm × 2 mm × 0.5 mm, the relationship of the *PV* in the chamber with time is obtained, as shown in Figure 4d. As the range of *PV* (difference between maximum and minimum) is no more than 4.6 × 10^−10^ Pa·m^3^, the leak rate is estimated to be under 1.02 × 10^−16^ Pa·m^3^/s. Although the vacuum degree of the microscale vacuum chamber is not good enough for some high-vacuum applications, the result shows that using monolayer graphene as lateral feedthrough could maintain a vacuum of 185 Pa and a leak rate of less than 1.02 × 10^−16^ Pa·m^3^/s for about two months, realizing a high sealing performance. In the future, a high-vacuum microscale chamber using a graphene electrode as lateral feedthrough could be realized by improving the bonding process to further improve the initial vacuum degree of the chamber.

## 4. Conclusions

In this paper, an on-chip microscale vacuum chamber with dimensions of π × 2 mm × 2 mm × 0.5 mm was fabricated, where a graphene electrode was innovatively used as a lateral electrical feedthrough. The monolayer graphene feedthrough shows a slight increase in resistance from 22.5 Ω to 31 Ω after anodic bonding, showing good electrical conductance. Benefiting from the atomic thickness of monolayer graphene, the leak resulting from the height difference between the electrode and the substrate surface was reduced, which effectively improved the sealing performance. The leak rate of Si/SiO_2_–glass bonding interface with a monolayer graphene feedthrough was measured to reach the measurement limit of the detector, namely less than 2 × 10^−11^ Pa·m^3^/s. For a bonded three-layer device with a microscale chamber, the pressure of the chamber was estimated as 185 Pa and had been maintained for more than 50 days. During this period, the leak rate was less than 1.02 × 10^−16^ Pa·m^3^/s, achieving a high sealing performance. In summary, the on-chip microscale vacuum chamber using graphene as lateral feedthrough is a reliable, simple and promising electrical feedthrough solution for vacuum electronic devices and other MEMS devices.

## Data Availability

Not applicable.
